# Does the Human Gut Virome Contribute to Host Health or Disease?

**DOI:** 10.3390/v15112271

**Published:** 2023-11-17

**Authors:** Grazia Pavia, Nadia Marascio, Giovanni Matera, Angela Quirino

**Affiliations:** Unit of Clinical Microbiology, Department of Health Sciences, “Magna Græcia” University Hospital of Catanzaro, 88100 Catanzaro, Italy

**Keywords:** human gut virome, gut immunity, enteric mucosal immunity, gut virota, virome–host interaction, gut virome–host immune axis, immune homeostatic mechanisms

## Abstract

The human gastrointestinal (GI) tract harbors eukaryotic and prokaryotic viruses and their genomes, metabolites, and proteins, collectively known as the “gut virome”. This complex community of viruses colonizing the enteric mucosa is pivotal in regulating host immunity. The mechanisms involved in cross communication between mucosal immunity and the gut virome, as well as their relationship in health and disease, remain largely unknown. Herein, we review the literature on the human gut virome’s composition and evolution and the interplay between the gut virome and enteric mucosal immunity and their molecular mechanisms. Our review suggests that future research efforts should focus on unraveling the mechanisms of gut viruses in human homeostasis and pathophysiology and on developing virus-prompted precision therapies.

## 1. Introduction

The gut microbiome comprises a large community of microorganisms (microbiota) and their collective genomes, metabolites, and proteins released in the gastrointestinal (GI) tract [1]. It is known that the generated products, such as short-chain fatty acids, interact with enteric mucosal host cells and influence physiological immune responses, protecting the host from infection and maintaining the function and morphology of intestinal epithelial cells [1,2]. The enteric microbiome includes different subsets, such as the virome (viruses), the mycobiome (fungi), the archaeome (archaea), and even some parasites, which diversify during individual growth [3,4,5,6,7,8,9]. In recent years, several studies have focused our attention on the human gut bacteriome’s characterization and functional role. It is currently considered a core “organ” regulating the balance between health and disease [10]. Indeed, when the intestinal microbial population undergoes a quantitative or qualitative change in composition (dysbiosis), this can result in an imbalance in homeostasis and the development of metabolic disorders and chronic diseases [11,12,13,14]. The recent development of highly sensitive metagenomic approaches and cutting-edge bioinformatic pipelines has shed light on the composition of commensal viral communities. These communities, in addition to bacteria, establish fundamental interactions with each other and the host immune system, thus collaborating in the maintenance of the health status and diversification of gut microbiota [6,10,15,16,17]. The human gut virome harbors eukaryotic and prokaryotic viruses that share lytic/lysogenic or latent life cycles, promoting their propagation and the evolution of microbiota composition. The enteric virome population includes (i) viruses that infect microbes (such as bacteria, fungi, and archaea), (ii) viruses that infect human cells, and (iii) plant viruses that are primarily derived from the environment and diets [6,18,19]. In addition, rare viruses, along with uncharacterized viral species, called “viral dark matter”, are also often reported [19].

However, it is unclear how the immune system actively recognizes and responds to the commensal human virome in the absence of classical inflammatory processes. Several viruses cause acute or chronic infection, either killing the host or being cleared by the immune system [12,20,21,22]. In many other cases, viruses coexist with their host as symbionts, either temporarily or for the duration of the host’s life, becoming beneficial to the host, providing protection from other infections and stimulating immunity. The paradigm of virus–host symbiosis is still open to debate. 

The present review summarizes recent knowledge on the human gut virome’s composition, evolution, and interactions and the responses between the gut virome and enteric mucosal immunity. Furthermore, we examine the molecular mechanisms responsible for recognizing the gut commensal virome in order to better understand how variations within the systemic and local gut viromes (commensal viruses) might shape the host’s immunophenotype. Based on recent findings, we searched for papers published in English for each period and included all types of papers, such as reviews, retrospective analyses, and experimental studies [23]. The mesh terms included the following keywords: “gut virome”, “gut immunity”, “enteric mucosal immunity”, “gut virota”, “gut virome-host interaction”, “gut virome-host immune axis”, and “gut immune homeostatic mechanisms”.

## 2. Human Gut Virome: Its Composition and Evolution

**Figure 1:**
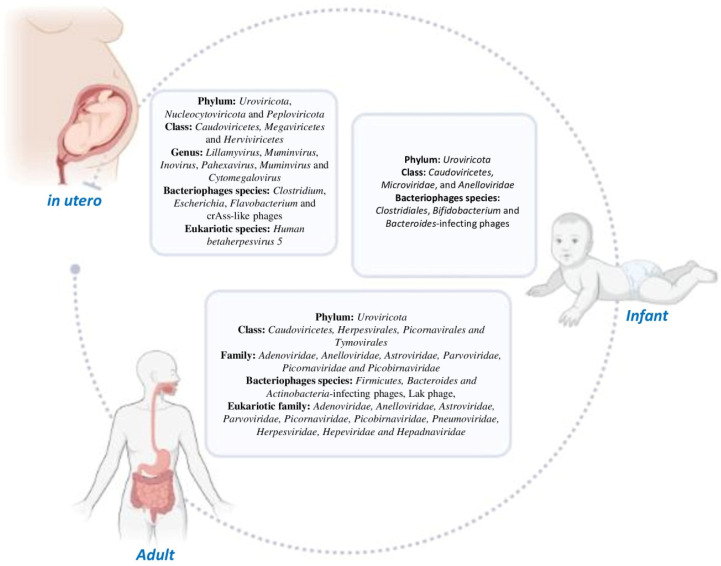
Composition and evolution of human gut virome composition from early life (in utero) to infancy and adulthood.

It is generally known that the microbiome, including the virome, begins to colonize the GI tracts of newborn infants immediately upon exposure to a non-sterile environment [24]. Large-scale studies establishing virome composition in utero are scant, and some of these [25] have been challenged both for containing large proportions of undefined viral populations and allowing the cross-contamination of biological reagents or samples [26,27,28,29,30]. However, with advances in metagenomic technologies, more evidence is suggesting the presence of microorganisms in the human placenta and in the *fetus* [7,31]. Enteric microorganism communities evolve dynamically during the first years of life, contributing to the maturation of the infant immune system [32]. During early life, gut microbiota undergo continuous changes that shape their longer-term composition and function [33]. The mode of birth [34,35], nutrition [36], maternal diet [37], and antibiotic exposure [38], can all impact the gut virus population, leading to the development of some pathological conditions in the *fetus* and later on in childhood, such as asthma, atopy, and congenital anomalies [39,40,41]. An infant’s gut virome changes rapidly during its early life until, at 2–3 years of age, the gut microbiota begins evolving into an adult-like structure [26,42,43]. A subsequent notable change was reported after 65 years of age, when the gut virome is subject to age-dependent variations in concomitance with the total microbiome of the GI tract [42,44]. In Figure 1, recent findings on the composition of virota and their lifetime evolution are summarized.

However, substantial portions of this ecological niche have not been thoroughly characterized. More recently, Liu X. et al., by combining metagenomic and virome-focused sequencing approaches, investigated the presence of viruses in fetal tissues, including in the small intestine, cecum, and rectum, obtained from second-trimester (12–22 weeks) elective pregnancy terminations [7]. They found that the fetal gut was not a sterile environment but rather has a low-abundance but metabolically rich microbiome [7]. Regarding the viral population, around 700 different viral species, including 130 species of bacterial viruses (bacteriophages) and 570 species of eukaryotic viruses, have been observed. The primary virus phyla identified were Uroviricota (dsDNA bacteriophages), Nucleocytoviricota (dsDNA viruses), and Peploviricota (Herpes viruses) in the small intestine/rectum, while only *Uroviricota* was found in the cecum. The most prevalent genera reported were *Lillamyvirus* (dsDNA bacteriophages), *Muminvirus* (dsDNA bacteriophages), and *Inovirus* (ssDNA bacteriophages) in the small intestine and *Pahexavirus* (dsDNA bacteriophages), *Muminvirus*, and *Lillamyvirus* in the rectum. Interestingly, no viral genera were detected in the cecum. Regarding the species of bacteriophages that can interact with bacteria to regulate bacterial composition, *Clostridium-*, *Escherichia-,* and *Flavobacterium*-infecting phages were the most prevalent in the small intestine/rectum, while among the eukaryotic viruses, *Human betaherpesvirus* 5 (also termed *Human Cytomegalovirus*) was the most abundantly observed in the rectum. Moreover, crAss-like phages were detected, which are not only the most abundant viruses known to exist in the adult human gut but also the most ubiquitous [7], suggesting that crAssphage is acquired in early life (Figure 1).

The first report on virome composition in the infant gut dates back more than a decade [45], but it has recently been reported that the infant virome is influenced by caesarean section (CS), milk [34], and pre-delivery prophylactic antibiotics [46]. Vaginal delivery (VD) generates more variability in virome components compared to CS, with a predominance of *Caudoviricetes* (dsDNA bacteriophages), *Microviridae* (dsDNA bacteriophages), and *Anelloviridae* (circular ssDNA viruses) [34]. Recently, 647 fecal samples from 1-year-old infants enrolled in the Copenhagen Prospective Studies on Asthma in Childhood 2010 (COPSAC2010) cohort [47] were characterized using a Next-Generation Sequencing (NGS) de novo assembly approach and classification [8,47]. In contrast to the adult gut, which is dominated by the virulent *Crassvirales* phage order, the researchers showed that a diverse and largely temperate group of phages dominate the infant gut virome. Most of the new viral family-level clades (VFCs) identified were previously unknown and belong to the *Caudoviricetes* viral class. In particular, temperate phages were found to be more prevalent and diversified in the 1-year-old gut virome, with the crAssphages being overshadowed by several previously undescribed viral clades. The volumes of typical *Bacteroides*-infecting crAssphages were lowered by previously unknown phage families infecting *Clostridiales* and *Bifidobacterium* [47]. Such a comprehensive taxonomic resolution of gut virome data will aid future research into translational viromics during infancy (Figure 1).

The most abundant viruses that colonize the adult GI tract are a collection of crAss-like phages [48,49], which are known to infect bacterial species belonging to the *Bacteroides* genus (e.g., *B. intestinalis* and *B. xylanisolvens*) [50]. Another two prevalent clades were Lak phages [51], Gubaphages [52], and *Flandersviridae* [53]. However, Nishijima S. and colleagues [54] recently analyzed the human gut viral profiles of 4198 uniformly phenotyped Japanese individuals (with an age range of 15–70 years old). In accordance with previous studies, the most frequent families of dsDNA bacteriophages found were *Siphoviridae*, *Podoviridae*, and *Myoviridae* in the order *Caudovirales*. Among bacterial hosts, Bacillota, with an incidence of 413, followed by Bacteroidota and Actinomycetota, were the preeminent phyla. Conversely, *Bacteroides*, followed by *Ruminococcus*, *Blautia*, and *Bifidobacterium*, were the most frequent genera infected by phages. It is noteworthy that, albeit at a relatively minor occurrence rate, phages infecting bacterial hosts linked to human health and disease, including *Klebsiella*, *Akkermansia*, and *Eggerthella*, were also observed [55,56,57]. Interestingly, the distributions of viral orders, such as bacterial phyla and genera, were reported to be equal in all the analyzed individuals. The proportion of virulent and temperate phages varied among the hosts investigated. The phages the infecting the genera *Odoribacter*, *Bacteroides*, and *Parabacteroides* were predominantly characterized as being virulent, while those targeting *Roseburia*, *Dorea*, and *Anaerostipes* were primarily temperate with a prevalence greater than 80% of the phage population. Age showed the strongest association with both the gut viral population and bacterial subsets amongst all the predictors correlated with higher gut microbiome variability (e.g., age, clinical factors, medications, and diseases) investigated [54].

The most abundant eukaryotic viral order in the intestinal mucosa is *Herpesvirales*, followed by *Picornavirales* and *Tymovirales* [58]. *Adenoviridae*, *Anelloviridae*, *Astroviridae*, *Parvoviridae*, *Picornaviridae*, and *Picobirnaviridae* are the most representative families, and they may provoke symptoms or long-term latency in healthy individuals [59]. Likewise, a wide-ranging set of enteric virus species with different tropisms were identified, including members of the *Pneumoviridae*, *Herpesviridae*, *Hepeviridae*, and *Hepadnaviridae* families, as well as viruses that infect insects and plants [59]. Additionally, endogenous retroviruses (ERVs), integrated in human DNA, induce a specific influence on human physiological processes [60]. In particular, ERVs are capable of triggering host immune responses and modulating the expression of specific genes contributing to tumorigenesis [15,60] (Figure 1).

## 3. Correlation between Human Virome and Bacteriome in the GI Tract

Recently, the largest single-cohort analysis on the human gut virome described novel viral clades in the GI tract and advanced insights regarding interactions between the virome and bacterial anti-viral genes, as well as factors strongly associated with the virome and bacteriome composition [54]. Through a comparative analysis, the corresponding researchers highlighted high degrees of correlation between the human virome and bacteriome. In particular, a high correlation of their diversities and an intricate association between the defense mechanisms of the bacteriome and virome diversity were shown. It has been proposed that phages within the human gut predominantly impact the composition of the bacteriome by causing bacterial lysis and incorporating themselves as prophages [54,61,62,63]. Researchers have found that the β-diversity of the virome is significantly higher than that of the bacteriome, suggesting that the virome is more specific to each individual than the bacteriome [54]. To further investigate their relationships, pairwise analyses of the relative abundance of each phage and its predicted host at the genus level among 4198 individuals were conducted [54]. Notably, it was observed that phages and host bacterial species tend to co-occur rather than exhibit mutual exclusivity in the human gut. Among the genera subjected to examination, *Megamonas*, *Escherichia*, *Prevotella*, and *Lactobacillus* displayed relatively stronger correlations with their respective phages compared to other genera. These higher correlations may be attributed to the greater prevalence of specialist phages infecting these genera, as opposed to genera like *Clostridium*, *Ruminococcus*, and *Tyzzerella*, which are more commonly targeted by generalist phages [54]. Prokaryotic species have evolved several defense mechanisms to protect themselves against the transfer of extra-chromosomial genetic elements and phage infections. CRISPR-Cas, restriction–modification, and abortive infection systems have been recognized as key defense elements of bacterial populations in the GI tract [64]. In this regard, Nishijima S. and colleagues investigated the correlation between the expression of these bacterial genes and gut virome composition [54]. Interestingly, the authors highlighted a strict link between defensive bacterial systems and the diversification of individual viral gut populations [54]. Other genes, such as integrase and spore germination proteins, the latter of which is associated with species in the Bacillota phylum, were also positively correlated with virome diversity [65].

There is still a significant portion of the gut virome, particularly with regard to RNA viruses, that still remains uncharacterized. More research with a special emphasis on targeting unknown viral species in the human gut should be undertaken to enable a better understanding of the role of this “dark viral matter” in human health and disease.

## 4. Advances in Viral Metagenomic Approaches

The study of the virome is relatively underdeveloped compared to the parallel assemblage of its bacterial counterpart due to the lack of a universally conserved viral gene, equivalent to the 16S rRNA gene in bacteria. The development of metagenomic approaches was a milestone in the study of virus populations harbored in the gut microbiome [16]. Despite the advanced technologies used in the study of the virome, molecular and computational approaches are not yet capable of characterizing the complete virus population in the GI tract, and our understanding of the composition of the gut virome remains incomplete [16]. The most common viral species characterized in the GI tract are dsDNA viruses (e.g., bacteriophages), while ssDNA, RNA, or multipartite genome viruses remain poorly represented [16,66]. This absence is at least partly due to genome extraction protocols favoring dsDNA [16,67]. Indeed, a new protocol, known as NetoVIR, was recently developed for commercial use to extract viral RNA genomes in addition to DNA genomes [68]. Moreover, the metatranscriptomic approach provides an interesting way to identify new ssRNA viruses, which can be extracted and sequenced in the same way as mRNA transcripts [69]. Furthermore, gut virome metagenomic analyses can often be influenced by the background contamination of other microbiota sequences due to the smallness of the viral genome subset. Therefore, several high-depth computational approaches have been designed, enabling the identification and removal of background microbiota contamination in silico [70,71,72,73]. Table 1 shows the description of the different steps of a viral metagenomics pipeline, including sample collection, storage and processing, sequencing, and bioinformatics analysis.

## 5. Mechanisms That Regulate Cross Communication between Commensal Viruses and Enteric Mucosal Immunity

**Figure 2:**
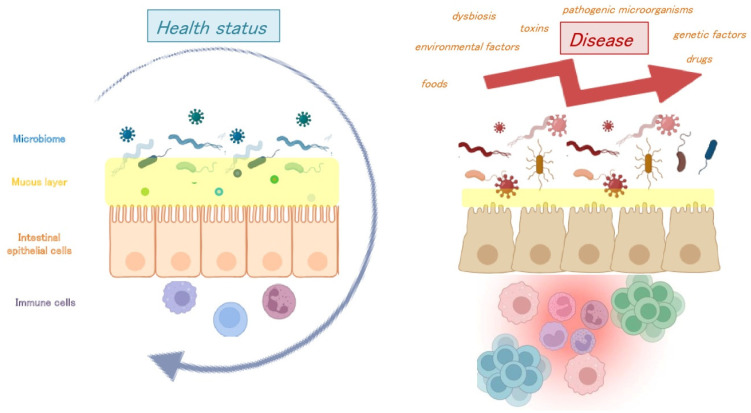
The interaction between intestinal epithelial cells, immune cells, microbiota, and metabolites. The strict “connection” between the above protagonists is necessary to create a balance in the protective immune response toward oneself and non-self entities (health status). Conversely, disruption of this equilibrium through genetic and/or environmental factors, such as foods, toxins, drugs, and pathogenetic microorganisms, is associated with intestinal epithelial damage and an altered immune response (disease).

The intestinal mucosa is composed of a complex plethora of cells that, through appropriate interactions, enable an immunologically tolerant environment necessary for the maintenance of homeostasis. The major components include intestinal epithelial cells, immune cells, microbiota, and metabolites [81,82]. Interaction between these components is necessary to create a balance in the protective immune response toward the host and non-host entities, respectively. Conversely, disruption of these constituents is associated with an altered immune response and may give rise to disease or abnormalities [11,13,14,15]. Maintaining the mucosal barrier is quite challenging since it is exposed to many genetic and environmental factors, such as foods, toxins, drugs, and microorganisms, including commensal viruses, which can induce several effects [82]. Disruption of such regulation contributes to inflammation, such as that occurring in Inflammatory Bowel Disease (IBD) [83], and, if severe, several metabolic and autoimmune diseases [84] (Figure 2).

### 5.1. Bacteriophages and Gut Mucosal Immune System

Bacteriophages are the most abundant viruses that colonize the enteric mucosa [61]. Like all other viruses, they exploit the cellular machinery of their bacterial host to replicate. These phages can be strictly virulent, targeting and killing bacteria (lytic cycle), or temperate, establishing symbiotic relationships with their host (lysogenic cycle) [61]. In these conditions, the virus integrates its genetic elements, enhancing the fitness and diversity of both the infecting phage and the bacterial cell [63]. Other phages remain sublethal in the hosts, releasing new virions [85]. Although phages do not directly cause human disease, as predators of bacteria, they could modulate the number, function, structure, and diversity of the commensal bacterial population [86]. Another type of phage colonizing enteric mucosa constitutes the filamentous phages (ssDNA), belonging to the sub-class *Inoviridae*. These bacterial viruses live in cooperative relationships with their host. In contrast with others, filamentous phages do not impose a burden on bacteria but provide support to improve their virulence by contributing to the spread of genes, much like cholera toxin [87].

As previously reported, bacteriophages play a crucial role in host defense by inducing lytic activity, removing pathogenic bacteria at sites of infection [88], and also interacting with mucus on the intestinal epithelial surface to enhance protective mucosal barriers, which prevents bacterial infection/translocation across the intestinal mucosa [89]. In this context, immunological cells may be triggered by the diversity of phages and induce responses. It has been reported that an imbalance in commensal phage populations can lead to changes in immune response, contributing to the development of several chronic immunological disorders, such as IBD, neurological disease, and obesity [83,84,90]. However, the mechanisms that regulate cross communication between mucosal immunity and bacteriophages (immunogenicity vs. tolerance) are still unknown. Many gaps in virome immunogenicity in health and disease remain unresolved; hence, research needs to be improved to clarify the “dialogue” between commensal viruses and mucosal immune cells.

At the mucosal luminal interface, phages interact with dendritic cells, also known as antigen-presenting cells (APCs), and transcytose in the epithelial cells or diffuse across impaired barriers to reach deeper tissues. Here, they are recognized intracellularly by pattern recognition receptors (PRRs), particularly Toll-Like Receptors (TLRs) such as TLR3 (dsRNA), TLR7 (ssRNA), TLR8 (ssRNA), and TLR9 (DNA); NOD-like receptors (NLRs) such as NLRPs; and RIG-I-like receptors (RLRs) such as RIG-I and MDA-5, or extracellularly by TLR2, triggering innate immune responses [91,92,93,94]. This interaction seems to be tightly controlled to avoid inflammatory immune responses against commensal viruses [94]. Usually, virus-activated APCs trigger an antiviral immune response via the release of different circulating mediators: interferon (IFN)-β, interleukin (IL)-6, IL-10, and IL-12 [95]. The activated lymphocyte T cells release IFN-γ and other products, triggering a B cell response contributing to the antiviral inflammatory process [95]. Whether phages can activate APCs in a similar manner remains unclear. Some researchers have reported that phages induce cytokine production via APCs in mouse models, stimulating Th1 cells to produce IFN-γ; others have suggested that phages have no inflammatory effect on APCs [93]. Miernikiewicz et al. and Bocian et al. demonstrated that purified T4 and A3/R ion exchange myoviruses did not affect the differentiation of APCs derived from human monocytes in vitro [96,97]. Similarly, Freyberger et al. demonstrated that different phage types may be differentially immunogenic [98]. The in vitro incubation of *S. aureus* myovirus K with APCs derived from the differentiation of human monocytes did not result in an activation of the immune response [98]. Indeed, it has been reported that the different activation of TLR9 on the apical cell surface or basolateral membrane promotes a non-inflammatory tolerogenic cellular immune response or an immune pro-inflammatory response, respectively [99]. 

**Figure 3:**
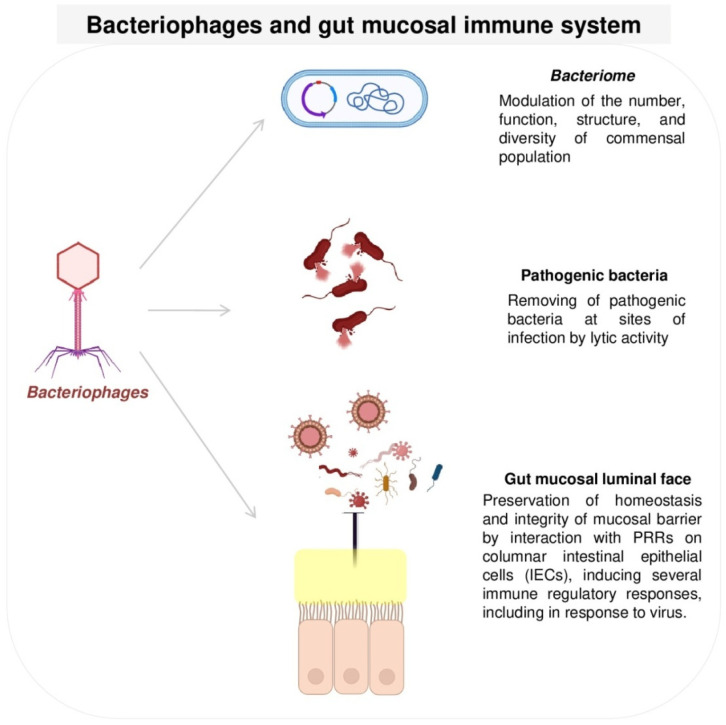
Bacteriophages and gut mucosal immune system modulation.

Commensal phages play a crucial role in maintaining homeostasis, regulating both the innate and adaptive host immunity [88]. In particular, it has been shown that these phages, by means of viral capsid Immunoglobin-like domains (Hoc proteins), bind the mucin glycoproteins on the intestinal mucous layer, providing an antimicrobial upfront defense against luminal bacterial pathogens [88]. Núñez-Sánchez et al., using an intestinal epithelium model, evaluated the therapeutic activity of gut bacteriophages against *Enterococcus feacalis* infection [100]. During intestinal colonization, *E. faecalis* adheres to and invades the intestinal epithelia, damaging tight junctions. Also, the *Enterococcus* phage A2 (a member of *Herelleviridae*) via virion-associated immunoglobin domains can, in turn, bind the mucus layer and translocate through the epithelium. Interestingly, in the presence of the *Enterococcus* phage A2, the *E. faecalis* population was reduced, and the integrity of the tight junctions was preserved. It was clearly evident that *Enterococcus* phage A2 reduced pathogen adhesion and translocation, in agreement with previous successful phage therapy against *E. faecalis* [101] (Figure 3). 

Although little is known about the specific mechanisms involved in phage-regulated innate and cellular immune responses, recent studies have reported that phages also have anti-inflammatory and immunomodulatory functions, which could be useful in different clinical conditions, such as those requiring treatment with allotransplantation procedures [102]. Bacterial infections were the primary risk for allograft recipients pre- and post-transplantation, followed by the toxic effects of immunosuppressive drugs required to prevent rejection and to induce immune tolerance [102]. Van Belleghem JD and colleagues have reported that human mononuclear cells infected by *S. aureus* and *P. aeruginosa* phages secrete IL-10 cytokine, regulating immune response [103]. This regulatory event, in addition to IL-10 production by B regulatory cells (Bregs), might contribute to the conversion of naive T lymphocytes to inducible Treg, preventing rejection and inducing immune tolerance [103]. The interest in phage therapy (PT), serving as a potential solution to modulating microbiota composition/diversity and as an effective therapeutic option for reducing the risk of infection and the toxic effects of immunomodulating drugs, has rapidly grown.

### 5.2. Eukaryotic Viruses and the Gut Mucosal Immune System

**Figure 4:**
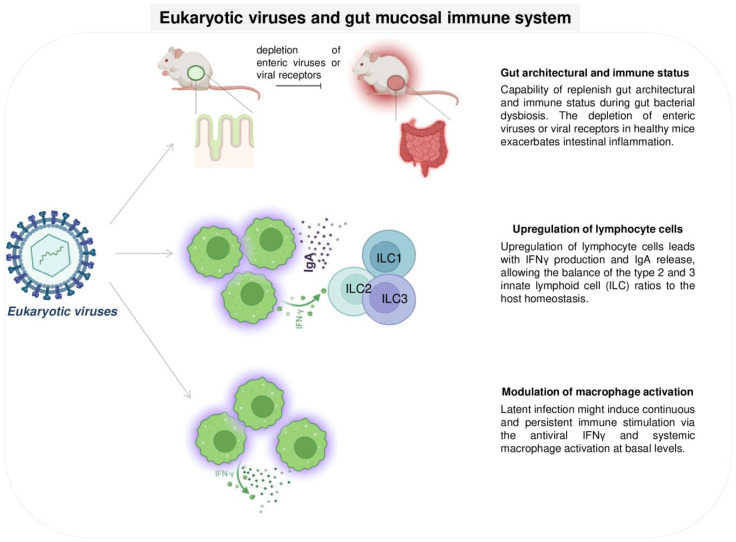
Eukaryotic viruses and gut mucosal immune system modulation.

Colonization by eukaryotic viruses is also crucial for the maintenance of gut homeostasis and host immunity. Several functional studies on mice have shown that the depletion of enteric viruses or viral receptors in healthy mice exacerbates intestinal inflammation, whereas treatment with viral ligands protects against disease [104]. Indeed, it has been shown that murine norovirus (MNV) is capable of restoring architectural and immune status during gut bacterial dysbiosis [105]. The researchers hypothesized that the mechanism regulating host immune homeostasis in the GI tract was correlated with lymphocyte cell upregulation paired with IFNγ production and IgA release, allowing balance of type 2 and 3 innate lymphoid cell (ILC) ratios [106]. Interestingly, this also occurred during infection or inflammation, highlighting the possible protective role that some enteric viruses may play [107]. Previously, Barton and colleagues [108] reported that eukaryotic viruses were beneficial to host homeostasis. It has been shown that mice latently infected with urine gammaherpesvirus 68 (MHV-68) or murine cytomegalovirus (mCMV) exhibit increased resistance to bacterial pathogens, such as *Listeria monocytogenes* and *Yersinia pestis*. It is probable that viral persistence leads to a continuous, low-level release of IFNγ, with a constant activation of macrophages. Indeed, Ingle and colleagues highlighted that a specific strain of murine astrovirus can play a crucial role in primary immunodeficiency. This viral complementation confers protection against enteric pathogens via IFN-λ signaling [108,109] (Figure 4).

An important goal for future research involves unraveling the precise mechanisms of action that occur in IFN downstream signaling in the models described above.

## 6. Gut Virome Dysbiosis and Transcriptional State in Healthy Host and Disease

The balance between beneficial and detrimental effects induced by the gut virome is mysterious. The precise mechanisms by which the virome provides protection are not well understood, and how they contribute to health and disease status has not been thoroughly clarified due to the lack of direct functional studies. However, we do know that prokaryotic and eukaryotic viromes have the capacity for immunomodulation based on reports of trans-kingdom interactions between bacteriophages and human immune cells [94]. Endogenous viral elements can regulate gene expression by encoding mRNAs for important functional proteins. For example, syncytiins, which are derived from endogenous retroviral genes, participate in placentation [110,111]. In this view of our metagenome, variations in the systemic virome may contribute to phenotypic variation by regulating immunophenotype rather than acting as pathogens. In several chronic, progressive immune-mediated conditions, such as IBD, it is well known that environmental factors, gut microbiota composition, genetic predisposition, and biological treatments contribute to the impaired immune response and disease evolution [112,113,114,115,116]. However, how the gut virome behaves in this condition is not yet well understood. A study of 28 subjects divided into patients with IBD and healthy control patients investigated whether the virome in general could affect host gene expression. The intestinal virome remained stable, which is in line with a previous report that suggested that the phage population, which makes up most of the intestinal virome, is highly stable and unique per individual [86,117]. Interestingly, although bacterial composition varies with worsening symptoms during an exacerbation, the phage population in longitudinal samples of patients with IBS was not affected during an exacerbation [118]. There is evidence of a correlation between the virome and 548 host genes at FDR < 1, representing multiple pathways related to immune response and infection, including toll-like receptor 2 (TLR2); CD4 molecule (CD4); interleukin 2 receptor gamma subunit (IL2RG); interleukin 6 receptor (IL6R); interleukin 3 receptor alpha subunit (IL3RA) and major histocompatibility complex, class II; DM beta (HLA-DMB); major histocompatibility complex, class II; DP alpha 1 (HLA-DPA1); and major histocompatibility complex, class II, DR alpha (HLADRA) genes [119]. Moreover, eukaryotic viruses that harbor in the GI tract may be markers of pathogenetic responses alone or in combination with environmental and genetic factors. This may be due to the latent persistent stimulation of enteric mucosal immunity, as already discussed above. Indeed, the continued stimulation of the gastrointestinal immune responses may evolve into chronic intestinal inflammation, such as IBD, with an exacerbated immune response and an uncontrolled inflammatory environment [117]. This theory has been supported by several studies [117,120], even if, so far, no evidence is available to support the causative role of the virome in the pathogenesis of chronic intestinal inflammation. Indeed, by studying in vivo models of dextran sodium sulfate (DSS)-induced colitis in mice with mutations in the Atg16l1 gene (alteration associated with Crohn’s disease), it has been demonstrated that MNV infection accelerates the development of colitis via virus-induced TNFα-dependent Paneth cell necroptosis [121,122], similar to that occurring in IL-10 KO mice, where mucosal inflammation is induced by MNVs and driven by the microbiota [123]. In addition, it has been reported that infection with enteric viruses, such as rotavirus, can accelerate the course of autoimmune diabetes in non-obese diabetic mice through the activation of lymphocytes in pancreatic lymph nodes via IFN signaling-I [124,125]. In the same way, extraintestinal viruses, such as Influenza A virus, can damage intestinal tissue through microbiota-mediated Th17 inflammation [104,126]. These insights shed light on the possibility that enteric virus infections trigger the pathogenesis of several diseases in genetically predisposed individuals.

## 7. Conclusions

Correlations between the human gut virome and the bacteriome have been highlighted, showing a high correlation in their diversity and an intricate association between the defense mechanisms of the bacteriome and the diversity of the virome. A greater understanding of the ecology and biology of the gut virome and the interactions between the kingdoms could lead to better therapeutic approaches allowing for the use of virome members to optimize health and well-being. The knowledge of the structure and function of the gut virome could help identify cause-and-effect relationships in the maintenance of health and their association with dysbiosis and various disease phenotypes. However, the study of associations between the intestinal virome and its interaction with host mucosal immunity is subject to technical limitations. The main one is the inability to resolve the identity of functional viral genes, i.e., viral sequences that do not align with any current viral sequence database. This may be due to highly divergent and highly evolving viruses as well as undiscovered viruses. The responses induced by various virus–microbiome and virus–host genome interactions likely alter the magnitude and function of the immune response to either the detriment or benefit of the host, leading to either the potentiation of or protection from disease. Human commensal viruses are clearly an important component of the holobiont that makes a major contribution to the health status of the host, yet the concept of a ‘healthy’ virome remains elusive and dynamic due to the inherent challenge of confirming that any virus will not induce disease under any conditions. Omics approaches for characterizing this “dark matter”, including metagenomics, meta-transcriptomics, and metabolomics, are particularly helpful in understanding the mechanisms by which the intestinal virome interacts with the host and could allow for a more comprehensive analysis of the gut virome.

Take-Home Messages

- Currently, it is unclear how the immune system actively recognizes and responds to the commensal human gut virome in the absence of classical inflammatory processes.
- The human gut virome is more specific to each individual than the bacteriome, becoming beneficial to the host through protection from other infections and the stimulation of immunity.
- Despite the advanced technologies used in the study of the virome, a significant portion of the gut virome, particularly RNA viruses, remains still uncharacterized.
- Bacteriophages are the most abundant viruses that colonize the enteric mucosa. These play a crucial role in host immune defenses, in the modulation of the commensal bacterial population, in preventing bacterial infection/translocation across the intestinal mucosa, and in inducing several immune regulatory responses, including in response to viruses.
- It has been shown that eukaryotic viruses in the GI tract are capable of (i) restoring gut architectural and immune status during gut bacterial dysbiosis and (ii) regulating host homeostasis, and (iii) it has been hypothesized that their latent infection might induce continuous and persistent immune stimulation via antiviral IFNγ and systemic macrophage activation at basal levels, protecting the host from infections.
- The precise molecular mechanisms by which the virome provides protection are not well understood, and how they contribute to health and disease statuses is not well clarified due to the lack of direct functional studies.
- More research with a special emphasis on targetting unknown viral species is necessary for characterizing this “dark matter” in order to understand the mechanisms by which the intestinal virome interacts with the host in human health and disease and to provide a more comprehensive analysis of the gut virome.

## Figures and Tables

**Table 1:** Different steps of a viral metagenomics pipeline, including sample collection, storage and processing, sequencing, and bioinformatics analysis.

Biological Sample Collection	Stool Sample	References
Storage	Temperature of −80 °C	[74]
	Specific viral media	
	Buffer	
Sample extraction protocol	Homogenization procedure	[68,75,76,77]
	Centrifugation and filtration	
	Sample concentration and viral enrichment	
	Host nucleic acid depletion via nuclease treatment	
	Random viral nucleic acid amplification	
High-deep sequencing	Sequencing platform	[16,69,78]
	Sequencing technologies (high-throughput short-read, paired-end reads, long-read and mate-pair read, de novo sequencing, etc.)	
	Sequencing depth	
Computational approach	Pre-processing quality control	[70,72,73,79,80]
	Alignment to remove potential contaminants in silico	
	Identification tools with integrated genomic international databases	
